# Transcriptome sequencing identified hub genes for hepatocellular carcinoma by weighted-gene co-expression analysis

**DOI:** 10.18632/oncotarget.9555

**Published:** 2016-05-23

**Authors:** Qi Pan, Xianli Long, Liting Song, Dachun Zhao, Xiaoyuan Li, Dewei Li, Min Li, Jinxue Zhou, Xia Tang, Hong Ren, Keyue Ding

**Affiliations:** ^1^ Key Laboratory of Molecular Biology for Infectious Diseases (Ministry of Education), Institute for Viral Hepatitis, Department of Infectious Diseases, The Second Affiliated Hospital, Chongqing Medical University, Chongqing, P.R. China; ^2^ Department of Pathology, Peking Union Medical College Hospital, Peking Union Medical College and Chinese Academy of Medical Sciences, Beijing, P.R. China; ^3^ Department of Medical Oncology, Peking Union Medical College Hospital, Peking Union Medical College and Chinese Academy of Medical Sciences, Beijing, P.R. China; ^4^ Department of Hepatobiliary Surgery, The First Affiliated Hospital of Chongqing Medical University, Chongqing, P.R. China; ^5^ Department of Hepatobiliary Surgery, Suining Central Hospital, Suining, Sichuan Province, P. R. China; ^6^ Department of Hepatobiliary Surgery, Henan Tumor Hospital, Zhenzhou, Henan Province, P.R. China

**Keywords:** hepatocellular carcinoma, transcriptome sequencing, weighted gene co-expression network, hub gene

## Abstract

Hepatocellular carcinoma (HCC) is one of the most common malignancies worldwide, and it remains a challenge to understand the genetic mechanisms underlying hepatocarcinogenesis. A global gene network of differential expression profiles in HCC has yet to be fully characterized. In the present study, we performed transcriptome sequencing (mRNA and lncRNA) in liver cancer and cirrhotic tissues of nine HCC patients. We identified differentially expressed genes (DEGs) and constructed a weighted gene co-expression network for the DEGs. In total, 755 DEGs (747 mRNA and eight lncRNA) were identified, and several co-expression modules were significantly associated with HCC clinical traits, including tumor location, tumor grade, and the α-fetoprotein (AFP) level. Of note, we identified 15 hub genes in the module associated with AFP level, and three (*SPX, AFP* and *ADGRE1*) of four hub genes were validated in an independent HCC cohort (*n*=78). Identification of hub genes for HCC clinical traits has implications for further understanding of the molecular genetic basis of HCC.

## INTRODUCTION

Hepatocellular carcinoma (HCC) is one of the most common malignancies worldwide, with the highest incidences occurring in East Asia and sub-Saharan Africa [1]. In China, HCC is the second leading cause of cancer deaths. Infection with chronic hepatitis B virus (HBV) remains the major etiological factor of HCC globally with more than one half of HCC patients being chronic HBV carriers [2]. Due to high mortality and poor 5-year survival rates [1], a better understanding of the genetic basis of HCC based on a more comprehensive approach will potentially provide novel strategies for its prevention and treatment.

The development of HCC is a complex biological process that involves the interaction of multiple genes [3]. With the advent of next-generation sequencing (whole genome/exome sequencing [4, 5] and transcriptome sequencing (RNA-seq) [6]), the genetic alterations underlying HCC at different molecular levels have been investigated. Multiple studies have characterized genome-wide mutational spectra of HCC [7–13], and identified at least 25 candidate driver genes with recurrent genetic alterations [14], including *TP53*, *CTNNB1*, and *ARID1A*. RNA-seq has identified new isoforms, fusion genes, and functional pathways that are altered in HCC [15–19]. In addition to protein-coding genes, long non-coding RNAs (lncRNAs) have recently been implicated in hepatocarcinogenesis, e.g., high expression of *HOTAIR* [20], *H19* [21], and *MALAT1* [22] have been observed in the liver cancer tissue. Although multiple genes involved in HCC have been identified, the relationship between gene expression and HCC clinical traits has been unclear.

In the present study, we performed a weighted gene co-expression network analysis (WGCNA) for HCC using mRNA- and lncRNA-seq data to investigate the association between differentially expressed genes (DEGs) and HCC clinical traits, e.g., tumor grade and the α-fetoprotein (AFP) level. We had the following aims: 1) investigate transcriptional patterns in liver cancer and cirrhotic tissues; 2) identify gene modules associated with HCC clinic traits and critical intramodular genes (i.e., hub genes); and 3) complement the characteristics of biological networks in the hepatocarcinogenesis.

## RESULTS

### Summary of RNA-seq data

We performed lncRNA and mRNA sequencing on nine pairs of liver cancer and adjacent cirrhotic tissues of hepatitis B virus-associated HCCs using the Illumina HiSeq^™^ 2000 (San Diego, CA) platform. RNA-seq generated 90 bp paired-end sequences and resulted in an output of a total 497 GB of raw sequence (approximately 13 GB per sample). On average, 54 and 57 million raw sequencing reads were obtained in lncRNA and mRNA sequencing, and approximately 94.1% and 93.9% of these raw reads were aligned to the transcribed database (http://genome.ucsc.edu/, hg19) (Table S1 and S2).

### Identification of lncRNAs in the liver cancer and cirrhotic tissues

**Figure 1:**
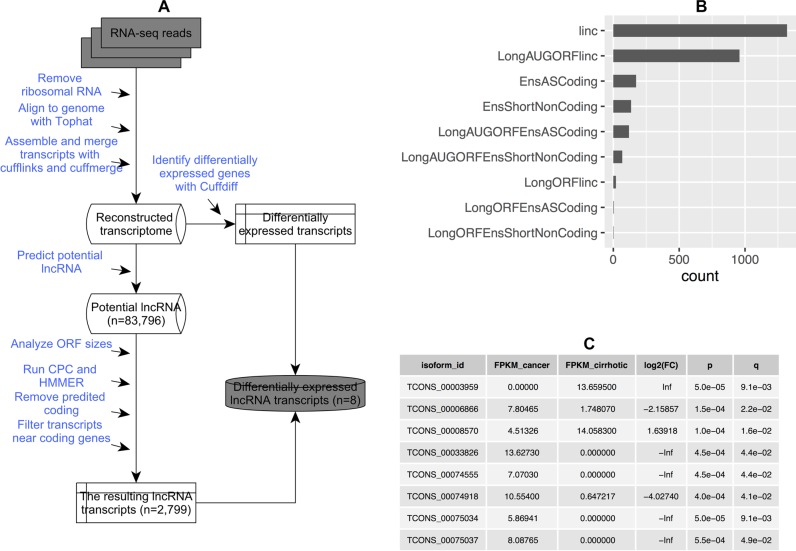
Identification of lncRNA (**A**) A pipeline for identifying and annotating lncRNA; (**B**) Number of distinct lncRNA transcripts identified in all samples. Linc: long intergenic non-coding transcript; LongAUGORFlinc: long intergenic non-coding transcript that contains open reading frames with in-frame codons enclosed within AUG and stop codons; EnsASCoding: an antisense transcript; EnsShortNoncoding: a precursor for small RNAs; LongAUGORFEnsASCoding: an antisense transcript that contains open reading frames with in-frame codons enclosed within AUG and stop codons; LongAUGORFEnsShortNoncoding: a precursor for small RNAs that contains open reading frames with in-frame codons enclosed within AUG and stop codons; LongORFlinc: long intergenic non-coding transcript that contains open reading frames; LongORFEnsASCoding: an antisense transcript that contains open reading frames; LongORFEnsShortNoncoding: a precursor for small RNAs that contains open reading frames; and (**C**) A list of significantly differentially expressed lncRNA transcripts.

We used a pipeline for lncRNA annotation from RNA-seq data (PLAR) to identify lncRNAs [23] (Figure 1A). The pipeline predicted 83,796 distinct transcript models that overlapped > 52% of the protein-coding genes (based on the RefSeq annotation in NCBI). After filtering predicted protein-coding transcripts and transcripts near coding genes, we finally obtained 2,799 non-coding RNA transcripts. The number of different types of non-coding RNA transcripts are shown in Figure 1B. In total, eight significantly differentially expressed lncRNA transcripts were identified, and none of these lncRNAs had been reported previously (Figure 1C).

### Significantly differentially expressed mRNA and lncRNA

**Figure 2:**
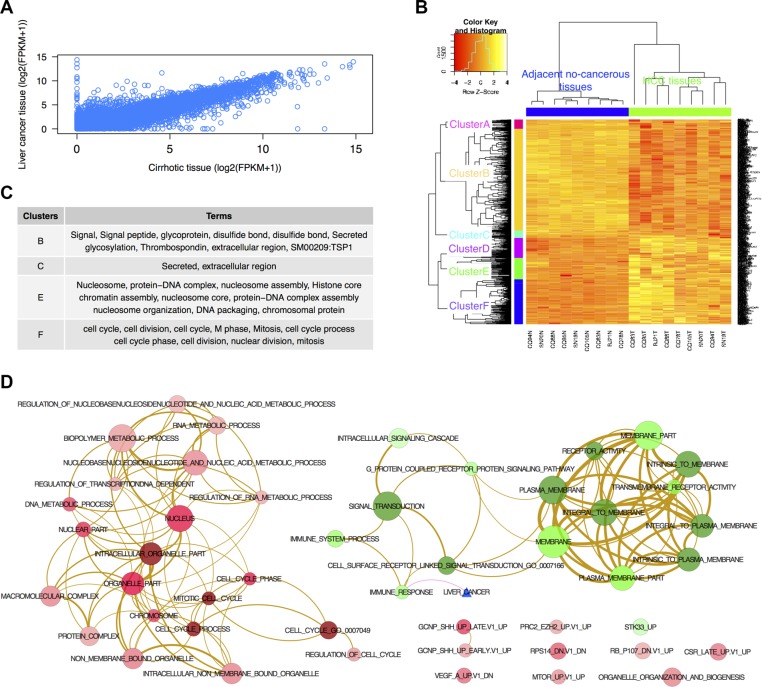
DEGs identified by RNA-seq (**A**) The correlation between genes expressed in the liver cancer and cirrhotic tissues; (**B**) Cluster analysis of significantly DEGs between nine liver cancer and cirrhotic tissues (fold change > 2 and FDR < 0.05). Rows represent genes, and columns represent samples. The dendrogram was generated from unsupervised cluster analysis of DEGs based on complete linkage and Pearson distances. The tree was cut by using the programme cutree for the hcluster at the h = max(hcluster$height)/1.2. Different coloured bars indicate different clusters. (**C**) GO terms for each cluster shown in B. (**D**) Network of enriched GO terms derived from the 747 DEGs between the liver cancer and cirrhotic tissues. Red nodes represent up-regulated terms, and green nodes represent down-regulated terms. The blue triangle represents the known liver cancer genes from the DiseaseHub database (http://zldev.ccbr.utoronto.ca/~ddong/diseaseHub/index.html). The color intensity in each node is proportional to enrichment significance. Purple edges indicate overlap between the liver cancer signature and the enriched gene sets, tan-color edges indicate overlap between two gene sets, and the edge width is proportional to the overlap size between the two nodes.

A total of 23,367 of genes were identified in nine liver cancer and cirrhotic tissues (Figure 2A). Using FPKM [24], we identified 747 significantly differentially expressed genes (DEGs) (fold change (FC) > 2, and false discover rate (FDR) < 0.05) between the liver cancer and cirrhotic tissues including 334 up-regulated and 413 down-regulated genes. For eight differentially expressed lncRNA transcripts, six up-regulated and two down-regulated genes were identified.

We estimated the statistical power for detecting significantly DEGs using ‘RnaSeqSampleSize’ [25] and ‘ssizeRNA’ [26]. The genes with minimun read counts > 10 across all individuals (*n* = 11,076) genes was used. We also estimated the distributions of gene read count and dispersions using our RNA-seq data as reference. Given a minimal FC of 2 (i.e., the effect size) and a FDR < 0.05, the statistical power to reject the null hypothesis that the population means of the two groups are equal is 0.697 (by an exact test [27]) using ‘RnaSeqSampleSize’ [25]. Given the same effect size and significance level, the achieved statistical power is 0.265 in nine pairs HCCs (by a paired *t*-test) using ‘ssizeRNA’ [26].

Cluster analysis of the DEGs produced six significant clusters (Figure 2B). Functional annotation for each cluster according to the gene ontology (GO) terms using DAVID [28] indicated that there were 57 significant GO terms (*p* < 0.05 and FDR < 0.05) including 22 cellular component terms, 30 biological process terms and five molecular function terms (Table S3). The top 10 GO terms for each cluster are shown in Figure 2C.

A gene set enrichment analysis (GSEA) for RNA-seq was performed to determine whether a set of genes defined a *priori* showed statistically significant, concordant differences between liver cancer and cirrhotic tissues. Five significant gene signatures (FDR < 0.05) were enriched in oncogenic signatures (Table S4). The β-catenin and Yes-associated protein conserved signatures were well known functional pathways involved in HCC [29, 30].

The enrichment map analysis overcomes the limitation of redundancy in the GO system [31]. A functional map was constructed using 47 enriched gene sets (30 up- and 17 down-regulated gene-sets) (Figure 2D). We noted that the up-regulated gene set was enriched in ‘nucleic acid metabolic process’ and ‘biopolymer metabolic process’ genes and that the down-regulated gene set was enriched in ‘signal transduction’ genes. We then tested whether the enriched gene sets were associated with the known liver cancer gene set (http://zldev.ccbr.utoronto.ca/~ddong/diseaseHub/index.html), which integrates data from multiple sources including OMIM (Online Mendelian Inheritance in Man), GAD (Genetic Association Database), HGMD (Human Gene Mutation Database), PharmGKB (Pharmacogenomics Knowledge Base), CGP (Cancer Genome Project) and GWAS (Genome Wide Association Studies). We found that the ‘immune response’ genes enriched in the known liver cancer gene set and two known liver cancer genes (*CCL5* and *CXCL12*) were associated with the enriched gene set (*p* < 10^−4^, Fisher's Exact Test).

### Gene co-expression network analysis of DEGs

**Figure 3:**
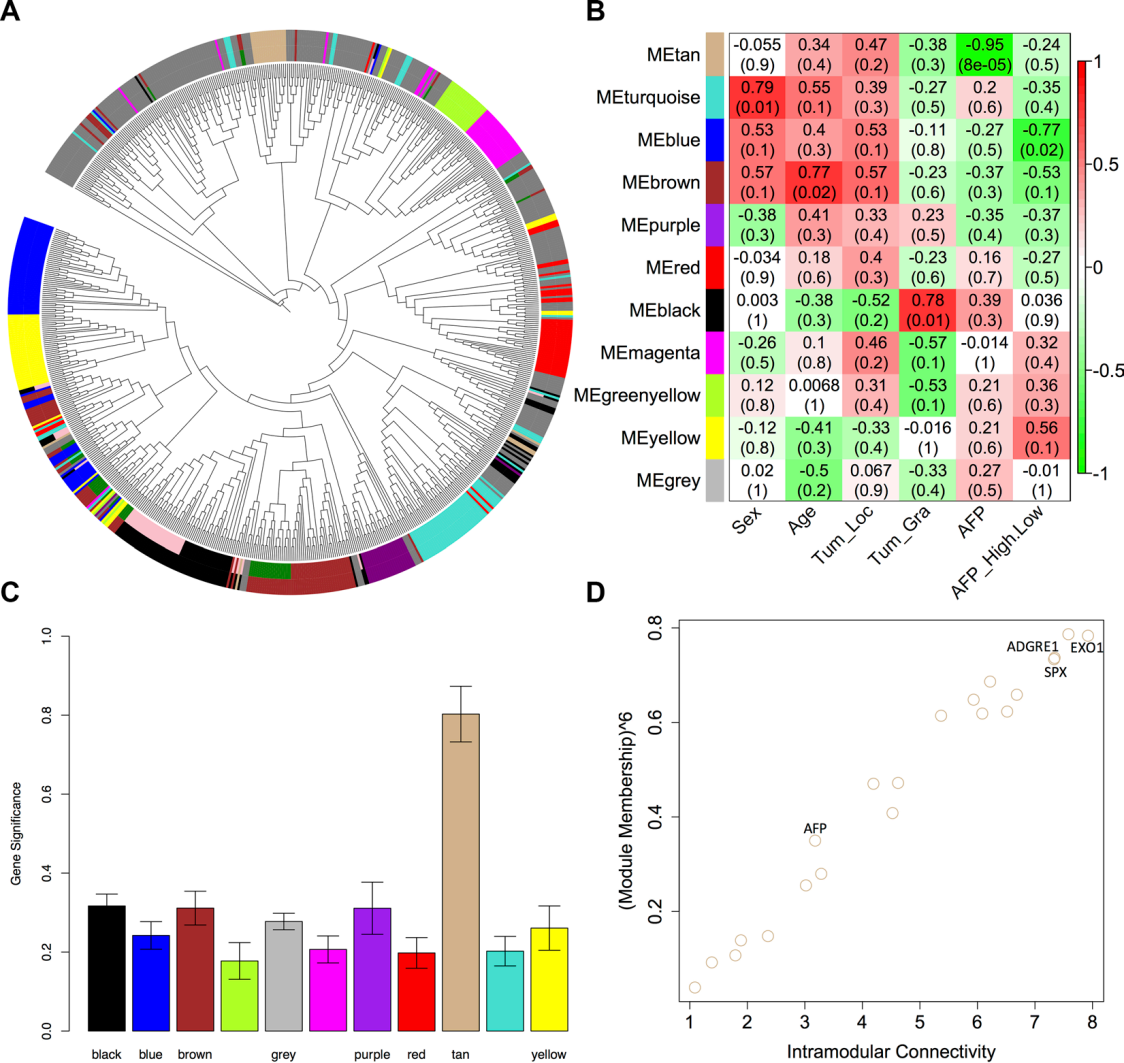
Identification of MEtan that was significantly correlated with AFP level (**A**) Dendrogram of all differentially expressed genes clustered based on a dissimilarity measure (1-TOM). Each line of the dendrogram corresponds to a gene. Circular tree shows hierarchical clustering of all differentially expressed genes. The inner ring shows the 13 modules identified using the dynamic cutting method with each gene color-coded based on module assignment. The outer ring shows the 11 modules identified using the merged dynamic method with each gene color-coded based on module assignment; (**B**) A heatmap of the correlation between module eigengenes and HCC clinic traits; (**C**) Distribution of the average gene significance and errors in the modules associated with AFP levels; and (**D**) Relationship between MEtan module membership measures and intramodular connectivity.

We decided to construct gene co-expression networks using the weighted gene co-expression network analysis (WGCNA). We extracted modules containing at least 15 genes by combining modules with eigengenes. The co-expression network contained 11 modules (Figure 3A), and the module sizes ranged from 21 to 94. However, 263 genes were not similarly co-expressed with other genes in the network (MEgrey), including eight lnc-RNA genes. The association between the modules and HCC clinical traits (i.e., sex, age, tumor location, tumor grade and the AFP level) were identified (Figure 3B). The correlation coefficient (*r*) of MEblack indicated that it was positively correlated with tumor grade (*r* = 0.78, *p* = 0.01) and that MEtan and MEblue were negatively correlated with the AFP level (*r* = −0.95, *p* = 8e−05) and the AFP high/low trait (*r* = −0.77, *p* = 0.02), where AFP > 25 ng/ml was considered to be high, respectively.

To verify the correlation between MEtan and the AFP level, a measure of module significance - the average gene significance of all of the genes in the module - was calculated. The distribution of the gene significance in all modules associated with the AFP level showed that MEtan had the highest mean gene significance (0.81) (Figure 3C), indicating that genes in MEtan may play an important role in affecting AFP level. Using Ingenuity Pathway Analysis (IPA^®^, http://www.ingenuity.com/products/ipa), we found that MEtan was enriched in genes involved in cell morphology (*p* = 0.03), embryonic development (*p* = 0.03) and hematopoiesis (*p* = 0.01) for molecular and cellular functions, and in the canonical eukaryotic pathways of CMP-N-acetylneuraminate biosynthesis I, phosphatidylcholine biosynthesis I, and mismatch repair.

Hub genes represent a series of genes that is significantly connected to a relevant module [32]. We identified the hub genes for the AFP level in MEtan based on the network property (the intramodular connectivity (IMC) and module membership (MM)) (Figure 3D). The top 15 hub genes were shown in Table 1 (FDR-adjusted *p* < 0.05), which explained 71% of the total variation in the module eigengene. Of the 15 hub genes, *SPX* was the gene most significantly associated with the AFP level (*q*. weighted value = 0.0025), and *EXO1* and *ADGRE1* exhibited the highest IMC (7.92) and MM (0.95), respectively. We also noted that the well-known HCC biomarker - *AFP* [33] - was associated with the AFP level (IMC = 3.18, MM = −0.84, and *q*.weighted = 0.025).

**Table 1:** Top 15 hub genes in MEtan module as defined by intramodular connectivity and module membership

Gene	q.Weighted	Intramodular connectivity	Module membership	Connections
*SPX*	0.0025	7.33	0.95	13
*ADGRE1*	0.0025	7.34	0.95	13
*EXO1*	0.0025	7.92	−0.96	12
*GPR88*	0.0025	7.58	0.96	12
*LOC645166*	0.0025	6.68	−0.93	12
*EGR3*	0.0028	6.51	0.92	13
*MT1X*	0.0034	6.08	0.92	11
*VARS*	0.0035	5.93	−93	12
*C19orf48*	0.0038	6.22	−0.94	12
*HMGA1*	0.0085	4.52	−0.86	11
*TMEM56*	0.0085	5.37	0.92	12
*ABCA8*	0.0086	4.62	0.88	9
*GNE*	0.0164	4.19	0.88	10
*AFP*	0.0254	3.18	−0.84	2
*RGL1*	0.0294	3.28	0.81	5

**Figure 4:**
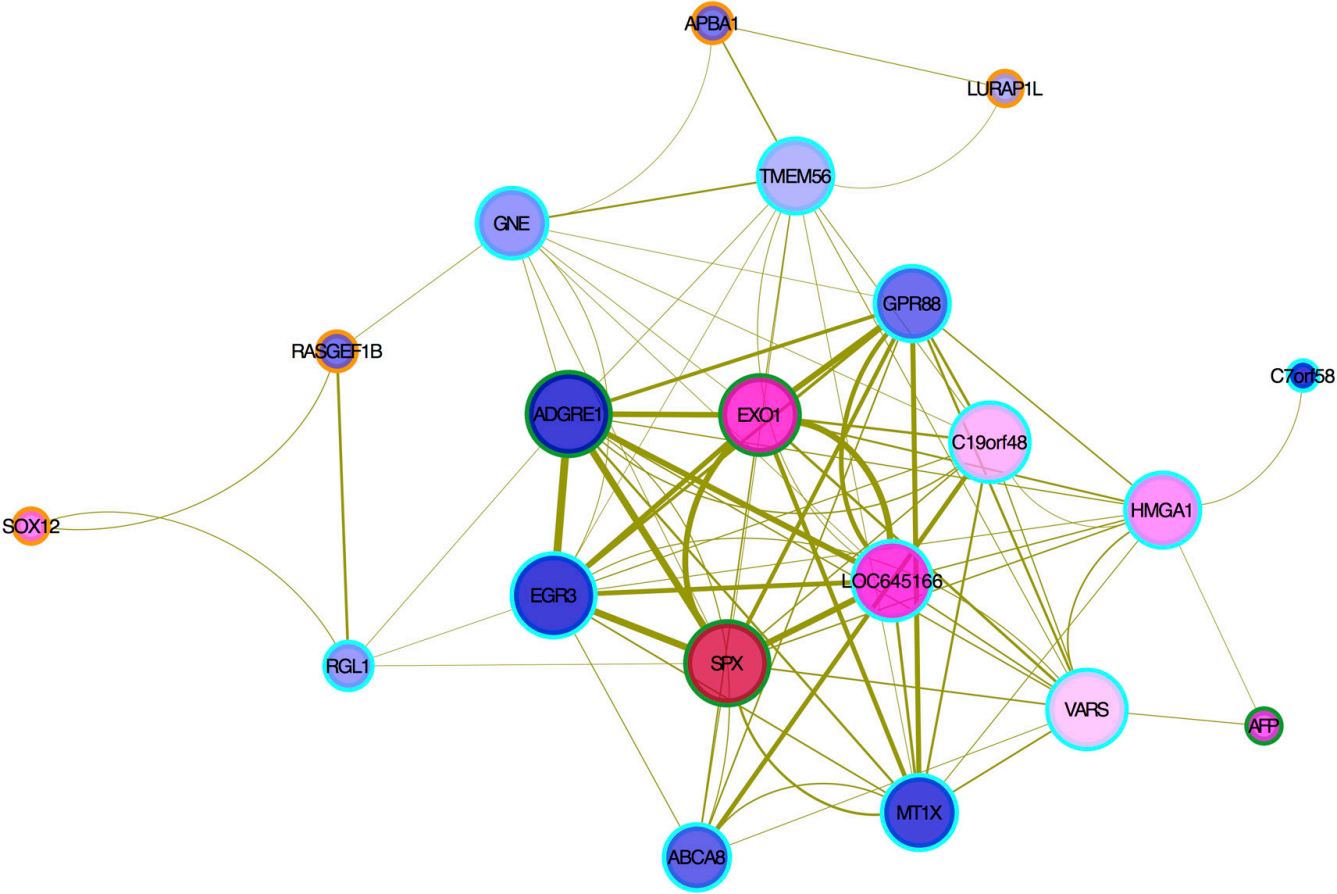
Hub gene interactions by co-expression pattern in MEtan module Node size is proportional to the degree of weighted connectivity. The edge width is proportional to the strength of connectivity between two nodes. The internal color in each node is based on the mean of log2 (FC) (up-regulated in red and down-regulated in blue): eight up- and 12 down-regulated genes in the co-expression network were noted. The outer ring color represents genes in MEtan (orange), non-validated hub genes (cyan) and validated hub genes (green), respectively.

A network visualization of the genes in MEtan associated with the AFP level is shown in Figure 4. A high interconnectivity among hub genes implies that the processes in which they are involved are potentially co-regulated. The most interconnected hub genes, with 13 strong connections (TOM > 0.1), were *SPX*, *EGR3* and *ADGRE1*. For example, *SPX* has strong interactions with *EXO1*, *ADGRE1*, *EGR3* and *GPR88*.

### Network preservation analysis and consensus analysis

**Figure 5:**
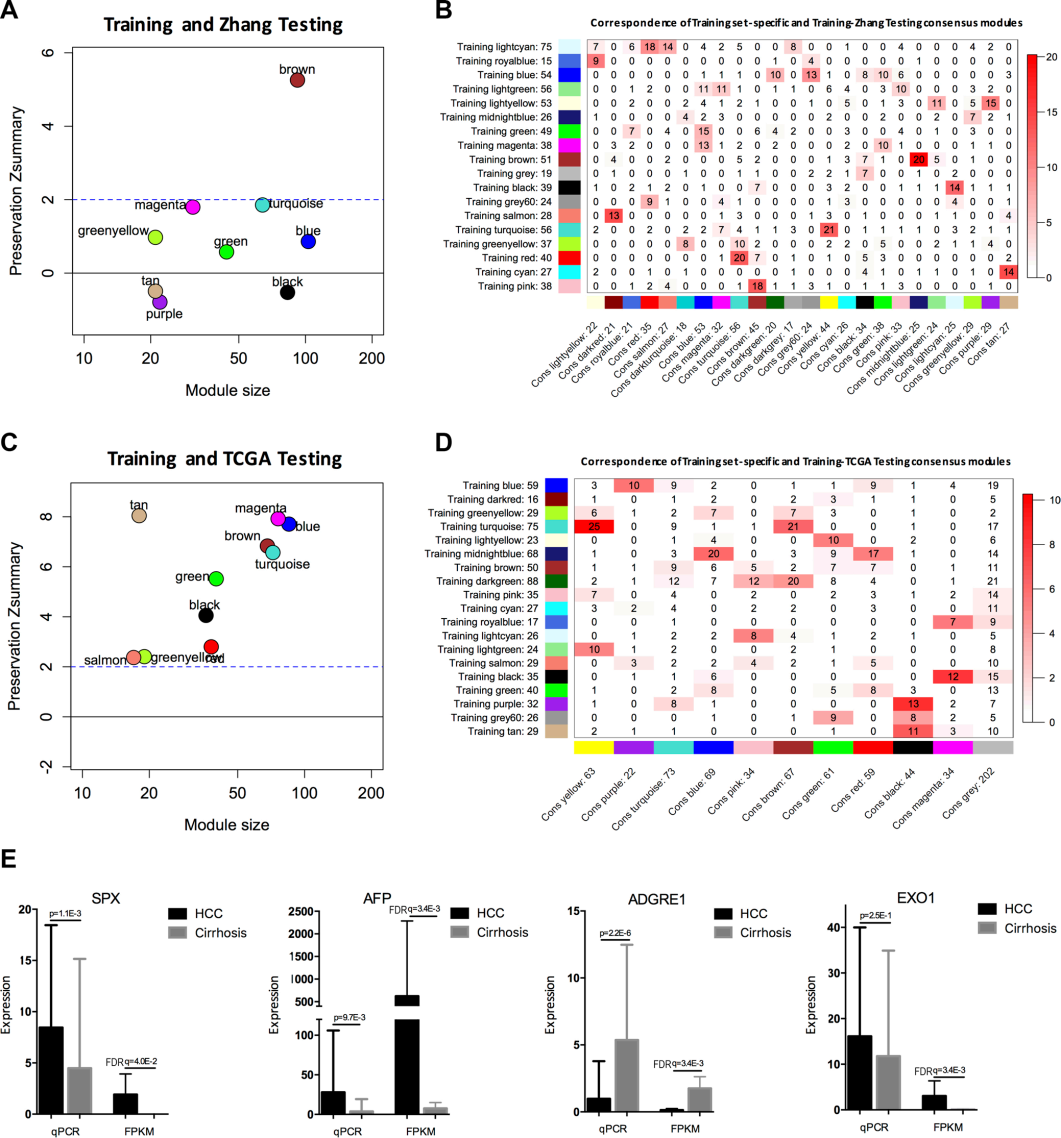
Validation of modules and hub genes The Zsummary (y-axis) as a function of the module size between the training and Zhang_testing_data (**A**), or TCGA_testing_data (**C**). The horizontal line shows the threshold of Zsummary = 2. Correspondence between the training data set-specific modules and training-Zhang_testing_data consensus modules (**B**) or training-TCGA_testing_data consensus modules (**D**) was shown. Each row of the table corresponds to one training data set-specific module, and each column corresponds to one consensus module. Numbers in the grid indicate the gene counts at the intersection of the corresponding modules. Colouring of the table denotes −log (*p*), with *p* being the Fisher's exact test for the overlap of the two modules. The stronger the red colour, the more significant the overlap is. The table indicates that most of the training data set-specific modules have a consensus counterpart. (**E**) Three of four selected hub genes were confirmed by qRT-PCR. Left: qRT-PCR; and Right: the FPKM values (mean sd) by RNA-seq; FPKM: Fragments Per Kilobase of exon per Million fragments mapped.

To validate the modules identified in the training data (i.e., our RNA-seq data), we assessed the preservation of modules in another two HCC RNA-seq data sets: a RNA-seq data set for 12 HCC patients (Zhang_testing_data, *n* = 12, accession no.: GSE63863) [34] and TCGA database (TCGA_testing_data, *n* = 50). We used a measure of intramodular connectivity preservation (i.e., Zsummary) to assess preservation [35]. In Zhang_testing_data, we found that MEbrown were moderately preserved (|Zsummary|>2), and the remainings modules were weakly preserved (|Zsummary|<2) (Figure 5A). In TCGA_testing_data, the modules showed highly preserved (|Zsummary|>8) (Figure 5C), e.g., Zsummary for MEtan related to AFP level was 8.04. These results suggested that the modules identified in the training data were reproducible in independent testing networks and there was no significant change in intramodular connectivity patterns. In addition, consensus analysis of associating the training data with two testing data sets respectively showed that most of the training set-specific modules have a consensus counterpart (Figure 5B and 5D). Our validation results suggested a similar module structure between the training and testing data.

We then used quantitative real-time PCR (qRT-PCR) to experimentally validate the expression levels of the hub genes in an independent HCC cohort (*n* = 78). As shown in Figure 5E, three out of the four selected genes (*SPX*, *AFP*, *EXO1*, and *ADGRE1*) were validated. The qRT-PCR and RNA-seq results were similar.

## DISCUSSION

In the present study, we conducted an expression profile analysis for nine liver cancer tissues and their matched cirrhotic tissues using transcriptome sequencing. We identified 747 DEGs and eight novel significantly differentially expressed lncRNA transcripts. The weighted gene co-expression network analysis identified a module (MEtan) related to AFP level and detected hub genes in MEtan. The identified modules were validated by preservation and consensus analysis in another two HCC RNA-seq datasets. We validated at least three hub genes (*SPX*, *AFP* and *ADGRE1*) in the module associated with the AFP level. To our knowledge, this study is the first one to integrate mRNA- and lncRNA-seq data to identify the hub genes related to HCC clinical traits.

We identified 747 DEGs in the liver cancer and cirrhotic tissues, which were categorized into six clusters. The DEGs were enriched in five oncogenic signatures. For example, the signatures of ‘BCAT_GDS748_DN’ and ‘BCAT.100_UP.V1_DN’ both source from β-catenin, a major effector of the canonical Wnt signaling pathway. The ‘CORDENONSI_YAP_CONSERVED_SIGNATURE’ is a YAP (Yes-associated protein)-conserved signature; YAP is a driving oncogene in HCC [30]. However, no significant consistency between the clusters and gene signatures was noted.

WGCNA is a powerful approach for investigating the mechanisms underlying the tumorigenesis because co-expressed genes are likely to be jointly involved in carcinogenesis [36]. The down-regulated of eigengenes in MEtan suggested that AFP is partially modulated by specific mRNAs. MEtan was predominantly enriched with cell cycle progression genes, playing an important role in hepatocyte proliferation. WGCNA identified lncRNA co-expression network to be associated with actively transcribed enhancers, which is involved in cell cycle deregulation and liver metabolism during HCC development [37].

We identified 15 highly connected hub genes in MEtan, including *ABCA8*, *AFP*, *EGR3*, *EXO1*, *HMGA1*, *MT1X* and *VARS*, which play roles as major regulators in cell-cycle regulation and cancer development. *ABCA8* is responsible for the transport of a variety of inflammatory mediators and lipids that have direct relevance to tumor progression in ovarian cancer [38, 39]. *EGR3* is the *bona fide* target for ESR and involved in the estrogen-signaling pathway in breast cancer cells [40]. *HMGA1* is involved in the carcinogenesis and invasiveness of HCC, which may be a potential prognostic marker [41]. By interacting with β-catenin, *HMGA1* positively regulates Wnt/ β-catenin signaling, leading to an increased formation of the β-catenin-TCF4 complex [42]. *EXO1* is an important nuclease involved in mismatch repair system that contributes to maintain genomic stability, modulate DNA recombination and mediate cell cycle arrest. A polyporphism in *EXO1* (K589E) was associated with increased risk of HCC development by influencing the activity of Exo1 protein [43].

We validated three of four selected hub genes in 15 hub genes (Figure 5E). Rucinski et al. [44] ascertained the role of spexin (*SPX*) in the regulation of cell proliferation (e.g., in adrenal gland cortex). The re-expression of *AFP* occurs in 50 to 80% of HCC patients during tumor progression, and the serum AFP levels play an important role in HCC diagnosis and the monitoring of responses to treatment [33]. Chen et al. [45] found several polymorphisms in the *AFP* promoter region may be pathologically significant in HCC. *ADGRE1* is an eosinophil receptor and a highly specific marker for eosinophils in humans [46]. Eosinophils reduce chronic inflammation linked to liver diseases in adipose tissue [47]. The association of *SPX* and *ADGRE1* with AFP level and HCC has not been reported previously. The underlying interactions between these hub genes affect AFP levels needs to be explored further.

Microarray data have been widely used in WGCNA for the identification of modules associated with cancer or intermediate traits [48–50]. However, several intrinsic limitations of microarray data should be noted; for example, each array contains only its own known genes, and microarray experiments are less reproducible [51]. In the present study, we performed WGCNA for HCC clinical traits using RNA-seq data, which has been suggested to be more reproducible [51].

There are several limitations in the present study. First, the number of samples used for RNA-seq was small, and a large sample size is needed to demonstrate the reliability of the results. However, preservation and consensus analysis of the identified modules in two independent HCC RNA-seq data sets indicated that modules in the training data were preserved in the testing data, especially in TCGA_testing_data. In addition, we validated at least three hub genes (of four) by qRT-PCR. Second, although we have identified a specific module (MEtan) that is associated with AFP level and have analyzed the possible molecular/cellular functions and canonical pathways in the module, there may still be other functions and pathways that were overlooked. Third, in the present study, we assumed one etiologic class (i.e., homogeneity) using HBV-associated HCC patients in RNA-seq. We cannot exclude the possibility that these hub genes may be involved in non-HBV associated HCC. Finally, we acknowledged that there is lack of functional validation of the hub genes.

In conclusion, our study identified differentially expressed mRNA and lncRNA between the liver cancer and cirrhotic tissues. A weighted gene co-expression network based on mRNA and lncRNA identified a module that was significantly associated with AFP level. Hub genes within the module may have crucial roles in HCC progression and may therefore be candidates for functional studies. Our study provided evidence that data mining of DEGs is an effective approach for the identification of novel genes associated with HCC clinical traits.

## MATERIALS AND METHODS

### Patients and tissue specimens

A total of 232 paired fresh-frozen tissue samples (the liver cancer and cirrhotic tissues) were collected from HBV-related HCC patients undergoing surgery in four hospitals (Table 2). All of the samples were immediately frozen in liquid nitrogen after surgical resection. Diagnosis of HCC for all of the cases was histologically confirmed by two independent pathologists at Peking Union Medical College Hospital. All of the tumour tissues were assessed by hematoxylin and eosin (HE) staining, and only tumour tissues with the percentage of tumor cells > 70% were used for analyses. Afterwards, the liver cancer and cirrhotic tissues of nine HCC patients were selected for RNA-seq. After pathological confirmation, 87 patients were used in the present study including nine pairs RNA-seq HCCs (Table 3) and 78 pairs HCCs used as an independent cohort for validation (Table S5).

**Table 2:** The number of liver cancer patients collected from four hospitals

Hospital	Recruited No.	Used No.
The First Affiliated Hospital of Chongqing Medical University (CQ)	130	59
Suining Center Hospital (SN)	50	17
Henan Tumor Hospital (HN)	50	10
Peking Union Medical College Hospital (PK)	2	1
**Total**	**232**	**87**

**Table 3:** The clinical and pathological features of the nine liver cancer patients

ID	Sex	Age (y)	Tumor size (cm)	% of tumor nuclei	HBV DNA	Tumor grade	HBV marker	AFP1 (ng/ml)
CQ78	M	59	4.6 × 4.2	95	< 1.3 × 10^4^	Low	HBsAg (+), HbeAg (−)	364
CQ83	F	31	13 × 12	70	Negative	Moderate	HbsAg (+), HbeAg (−)	36243
CQ88	M	44	11 × 10	90	1.5 × 10^6^	Moderate	HbsAg (+), HbeAg (−)	265196
CQ94	M	46	4 × 3	80–90	Negative	High	HbsAg (+), HbeAg (+)	8
CQ95	M	50	6 × 6	60	9.6 × 10^3^	Moderate	HbsAg (+), HbeAg (−)	147
CQ105	M	59	2.4 × 2.2	90	6.5 × 10^6^	Moderate	HbsAg (+), HbeAg (+)	9
SN01	F	46	3.5	90	3.3 × 10^6^	High	HbsAg (+), HbeAg (+)	1210
SN02	M	57	5.8 × 4.9	90	Negative	High	HbsAg (+), HBeAg (−)	2
BJ21	F	62	6.8 × 5.9	90	NA	High	HBsAg (+), HBeAg (−)	16189

1 AFP = α-fetoprotein.

The collection of human samples and the protocols for the investigations were approved by the an Institutional Review Board (IRB) in Peking Union Medical College Hospital, The First Affiliated Hospital of Chongqing Medical University, Henan Tumor Hospital, Suining Center Hospital and The Second Affiliated Hospital of Chongqing Medical University. The patient provided written informed consent in this study.

### cDNA library preparation and RNA-sequencing

Total RNA was extracted from nine HCC and cirrhotic tissues. We performed whole transcriptome sequencing for mRNA and lncRNA, as described in our previous study [52]. All sequencing was carried out at the Beijing Genomic Institute at Shenzhen (BGI-Shenzhen, Shenzhen, China). A detail description of the library preparation and sequencing is provided in the Supplementary materials.

### RNA-seq data analysis

We used a previously described protocol [53] to perform differential gene expression analysis. We used Tophat [54] to map the RNA-seq reads to the genome. Alignments were used as input for Cufflinks [55] for transcriptome reconstruction. The reconstructed transcriptome from all of the samples were merged using CuffMerge. Expression levels in each sample in Fragments Per Kilobase per Million reads (FPKM) units were quantified using CuffDiff [55]. Finally, we used cummeRbund [56] for further analysis. All programs were used with default parameters.

### Identification of lncRNA

We used a Pipeline for lncRNA annotation from RNA-seq data (PLAR) to identify lncRNAs [23] (Figure 1A). A detailed description was shown in Supplementary materials.

### Gene set enrichment analysis (GSEA) and enrichment map analysis

GSEA [57], which utilizes the gene rank derived from differential expression, is a computational method that determines whether a priori defined set of genes shows statistically significant, concordant differences between two biological states. An enrichment map organizes gene sets in a more intuitive way and is implemented in Cytoscape network analysis environment [31, 58]. Gene-sets derived from DEGs were enriched and filtered for significance (*p* < 0.05, FDR < 0.05). An enrichment map places similar significant gene sets near each other, resulting in a more concise global view of enriched biological functions. Overlap between significant gene sets is computed according to the overlap coefficient. The overlap between the DEGs found in the present study and the known liver cancer gene set was scored (Fisher's exact test, nominal *p* < 10^−4^) (http://baderlab.org/Software/EnrichmentMap).

### Weighted gene co-expression network analysis (WGCNA)

WGCNA is a statistical approach for the construction of gene modules within a network based on correlations between RNA expression profiles [36]. Considering that the WGCNA was nearly a scale-free topology, the weighted coefficient β was selected based on the scale-free topology criteria, allowing for a maximal correlation coefficient. The adjacency coefficient α was computed using a power function (α_mn_ = power (S_mn_ β) = 1S_mn_ 1^β^), which measures correlation strength between two genes. The adjacency matrix was created based on α, which was subsequently transformed into a topological overlap matrix (see Supplementary materials). A topological overlap measure (TOM) was calculated, which assessed gene interconnectedness.

### Identification of clinical significant modules

Genes were hierarchically clustered using the dissimilarity coefficient as the distance measure. We assigned modules containing at least 15 genes by using a mixed dynamic tree-cutting algorithm criterion, which was used to identify modules whose expression profiles were similar and then and merge them into new modules defined as merged dynamic modules. Module eigengenes associated with clinic traits were then used to calculate a correlation coefficient (*r*) between each module and an HCC clinic trait. The module significance is defined as the average gene significance in a module, and the gene significance is defined as log(*p*), where *p* denotes the significance from the *t*-test for the identification of differential expression between two groups. High module significance values denote strong associations with a clinic trait.

### Hub gene analysis

Genes with the highest degree of connectivity within a module (i.e., centrally located genes of co-expressed genes) are termed as ‘hub genes’ and are expected to be drivers required for signaling pathway of essential cellular function [36, 59]: ${\text{k}}_{\text{i}}=\sum_{\text{j}\neq \text{i}}^{\text{n}}{\text{a}}_{\text{ij}},\text{i},\text{j }\epsilon$ Module *q*, where k_i_ = intramodular connectivity of gene *i*, and a_ij_ = adjacency between genes *i* and *j* [35]. Alternatively, it may also be defined as genes with high module membership [35, 60]: MM_i_^(q)^ = cor (x_i_, E^(q)^), where MM_i_ = module memebership of gene *i* (in module *q*), x_i_ = expression profile of gene *i*, and E^(q)^ = module eigengene of module *q*. Both definitions were used to identify the hub genes of module associated with AFP level.

### Network preservation and consensus analysis

Module preservation between our HCC data (the training data) and the testing data [34] was assessed using network preservation statistics [35]. Module density-based and connectivity-based statistics were used to assess module reproducibility [35]. In this comparison, Zsummary, a *Z* statistic representing a weighted summary of module density and connectivity measures was computed for each module. The Zsummary score was used to evaluate module preservation; >8 indicating strong preservation, and 2–6 means moderate preservation [35]. In addition, we performed consensus analysis of the training and testing data, which related training data modules to the consensus modules and calculated the overlaps of each pair of training-consensus modules.

### Quantitative real-time PCR (qRT-PCR)

We used real-time quantitative PCR (Bio-Rad^®^, Hercules CA) to validate four hub genes that affected AFP level. The genes were selected by their significance levels and functional relevance. Paired *t*-tests were used to test for significance. A detailed description of the qRT-PCR method is presented in the Supplementary materials.

## SUPPLEMENTARY MATERIALS AND METHODS, REFERENCES, FIGURES AND TABLES


