# Supplementary material for: Transcriptome sequencing identified hub genes for hepatocellular carcinoma by weighted-gene co-expression analysis
Source: Oncotarget. 2016 May 23;7(25):38487–99. doi: 10.18632/oncotarget.9555 (PMC5122405; doi:10.18632/oncotarget.9555)
Supplement: Supplementary file 1 [file oncotarget-07-38487-s001.pdf]

Transcriptome sequencing identified hub genes for hepatocellular carcinoma by weighted-gene co-expression analysis

Supplementary Materials

MATERIALS AND METHODS

cDNA library preparation and RNA-sequencing

After extracting the total RNA from HCC and cirrhosis tissues, mRNA and non-coding RNAs were enriched by removing rRNA from the total RNA. The mRNA and non-coding RNAs were fragmented into short fragments (about 200~500 nt), then the first-strand cDNA was synthesized by random hexamer-primer using the fragments as templates, and dTTP was substituted by dUTP during the synthesis of the second strand. Short fragments were purified and resolved with EB buffer for end reparation and single nucleotide A (adenine) addition. After that, the short fragments were connected with adapters, then the second strand was degraded using UNG (Uracil-N-Glycosylase) finally [1]. After agarose gel electrophoresis, the suitable fragments were selected for the PCR amplification as templates. During the QC steps, Agilent 2100 Bioanalyzer and ABI StepOnePlus Real-Time PCR System were used in quantification and qualification of the sample library. As last, the cDNA library of mRNA and lncRNA was sequenced using Illumina HiSeq™ 2000 at Beijing Genome Institute. A detail description of cDNA library preparation and RNA-seq was shown in our recent study [2].

Identification of lncRNA

We used a pipeline for lncRNA annotation from RNA-seq data (PLAR) to identify lncRNAs [3] (Figure 1A). The pre-process of RNA-seq reads includes removing ribosomal RNA in comparing with mRNA. We used the Java program suit in the PLAR package to predict potential lncRNA. In the multiple filtration steps, we removed multi-exon transcripts having exonic sequences < 200 bases, or that were expressed at FPKM < 0.1 in all samples. Following analysis of 3P-seq data [4], only those single-exon cufflinks models with exonic length > 2,000 nt and an FPKM > 5 in at least one sample were retained. Transcripts that 50% of their exonic sequences annotated a single repeat were also removed. A transcript that overlapped the coding sequence of a protein-coding gene by at least one base and overlapped any of its exonic sequence by at least 100 nt was designated as protein-coding. A single-exon transcript contained within an intron of a protein-coding gene on the same strand was annotated as "intron contained". Transcripts overlapping on the other strand at least one base of the coding sequence of a protein-coding gene were considered as "antisense" transcripts and those overlapping on the same strand a small RNA gene where considered "small RNA primary transcripts".

Two methods were used for discovering protein-coding potential: CPC [5] and HMMER [6]. CPC was applied to repeat-masked transcript sequences, using the RefSeq database of protein sequences (only "NM_" entries). HMMER [6] was applied to repeat-masked transcripts translated in all three possible frames using the Hidden Markov Models (HMMs) of protein domains from the Pfam-A and Pfam-B databases [7]. Any transcript with a Pfam domain prediction with E < 0.001 was considered coding. Transcripts were designated as coding if their exons overlapped a predicted protein-coding element with $p < 10^{-4}$ by at least 10 bases. This filter was not used for antisense lncRNAs. Any gene that contained an isoform reported as coding by one of those programs was designated as "predicted coding". Transcripts proximal (within 500 nt for multi-exon or 2 Kb for single-exon) to an annotated or reconstructed protein-coding gene on the same strand were excluded. Transcripts that overlapped any annotated pseudogenes (http://pseudogene.org) were removed. We removed transcripts that started or ended within 500 nt of a protein-coding gene (2,000 nt for single-exon transcripts). We also removed single-exon transcripts that overlapped a multi-exon transcript by at least 50% of their exonic sequence.

Gene set enrichment analysis (GSEA)

Genes were scored for differential gene expression (*t*-test) by comparing liver cancer specimens and matched cirrhotic specimens. GSEA was used to find enriched GO gene-sets in up- or down-regulated genes. GSEA was run using gene sets from diverse public sources (described below). Small (< = 15 genes) gene sets were removed because these are more likely to appear significantly by chance alone. Large (> 500 genes) gene sets were removed because they are typically too general to usefully interpret. Filtering has the added benefit of reducing the problem of false discovery by multiple testing. For each analysis, 1000 gene set permutations were used to compute a false-discovery rate.

Weighted gene co-expression network analysis (WGCNA)

The WGCNA includes the following steps: 1) Gene clustering tree was created based on gene expression similarity matrix using 747 significantly differentially expressed mRNA and eight lncRNA. 2) Pruning the less connected nodes, the branch with the maximal gene was retained for subsequent analysis based on the dendrogram height. Considering that the WGCNA was close to scale-free topology, the weighted coefficient β was selected based on the scale-free topology criterions, allowing for maximal correlation coefficient. The β value was set as weighting coefficient only when the correlation coefficient between log (k) and log (p (k)) reaches 0.8, where p (k) is the proportion of nodes with connectivity k. Here, we selected the $\beta = 18$ so that the initial correlation coefficient reached 0.8.

The adjacency coefficient α was computed using a power function ($\alpha_{mn} = power\ (S_{mn}\ \beta)) = |S_{mn}|^{\beta}$), which measures correlation strength between two genes. Based on the adjacency coefficient, the adjacency matrix was created. The adjacency matrix was computed as: $\omega_{mn} = (l_{mn} + \alpha_{mn}) / (min\ (k_m, k_n + 1 - \alpha_{mn})$, where $S_{mn} = |\ cor\ (m, n)|$ represents expression correlation coefficient, $l_{mn} = \sum\mu\ \alpha_{m\mu}\ \alpha_{n\mu}$ represents the sum of products of the adjacency coefficient of the nodes connected to both m and n. $k_m = \sum_{\mu} \alpha_{m\mu}$ represents the sum of the adjacency coefficients of the nodes only connected to m. $k_n = \sum_{\mu} \alpha_{n\mu}$ represents the sum of the adjacency coefficient of the nodes only connected to n. $\omega_{mn} = 0$ if two nodes are not connected, and do not share any neighbors. The dissimilarity between two nodes was calculated as the formula $d_{mn}^{\omega} = 1 - \omega_{mn}$. After adjacency parameter was determined, the correlation matrix was transformed into an adjacency matrix. The adjacency matrix was subsequently transformed into a topological overlap matrix.

REFERENCES

1. Parkhomchuk D, Borodina T, Amstislavskiy V, Banaru M, Hallen L, Krobitsch S, Lehrach H, Soldatov A. Transcriptome analysis by strand-specific sequencing of complementary DNA. Nucleic Acids Res. 2009; 37:e123.
2. Ding K, Wu S, Ying W, Pan Q, Li X, Zhao D, Li X, Zhao Q, Zhu Y, Ren H, Qian X. Leveraging a Multi-Omics Strategy for Prioritizing Personalized Candidate Mutation-Driver Genes: A Proof-of-Concept Study. Sci Rep. 2015; 5:17564.
3. Hezroni H, Koppstein D, Schwartz MG, Avrutin A, Bartel DP, Ulitsky I. Principles of long noncoding RNA evolution derived from direct comparison of transcriptomes in 17 species. Cell Rep. 2015; 11:1110–22.
4. Jan CH, Friedman RC, Ruby JG, Bartel DP. Formation, regulation and evolution of Caenorhabditis elegans 3'UTRs. Nature. 2011; 469:97–101.
5. Kong L, Zhang Y, Ye Z-Q, Liu X-Q, Zhao S-Q, Wei L, Gao G. CPC: assess the protein-coding potential of transcripts using sequence features and support vector machine. Nucleic Acids Res. 2007; 35:W345–9.
6. Durbin R, Eddy SR, Krogh A, Mitchison G. Biological sequence analysis: probabilistic models of proteins and nucleic acids. 1998;
7. Finn RD, Bateman A, Clements J, Coggill P, Eberhardt RY, Eddy SR, Heger A, Hetherington K, Holm L, Mistry J, Sonnhammer ELL, Tate J, Punta M. Pfam: the protein families database. Nucleic Acids Res. 2014; 42:D222–30.

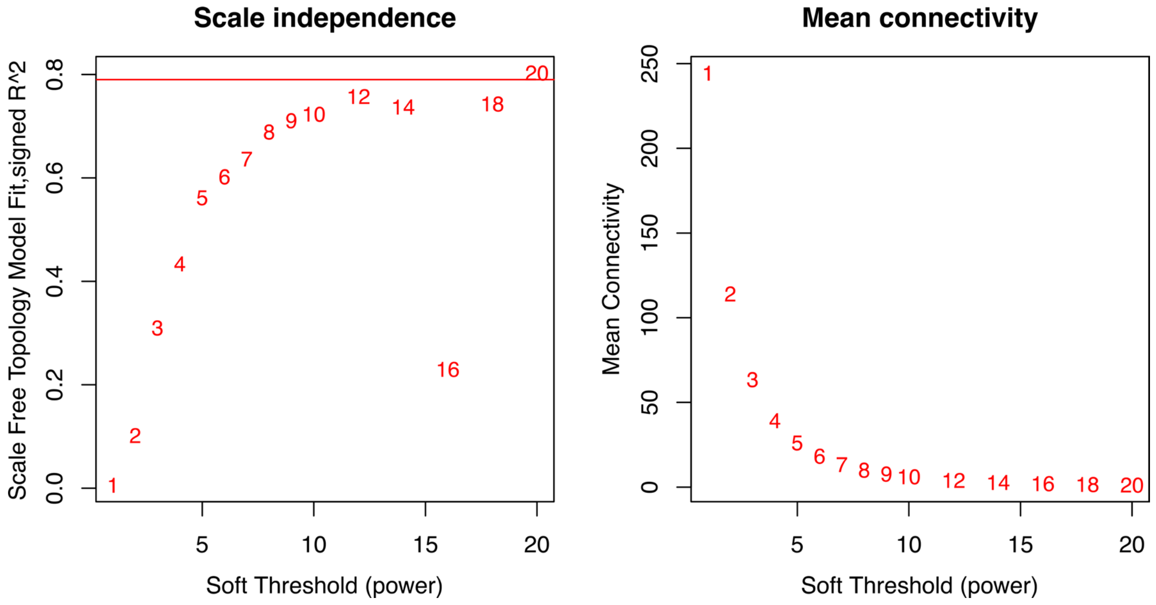

Supplementary Figure S1: Analysis of network topology for various soft-thresholding powers. The left panel shows the scale-free fit index (y-axis) as a function of the soft-thresholding power (x-axis). The right panel displays the mean connectivity (degree, y-axis) as a function of the soft-thresholding power (x-axis).

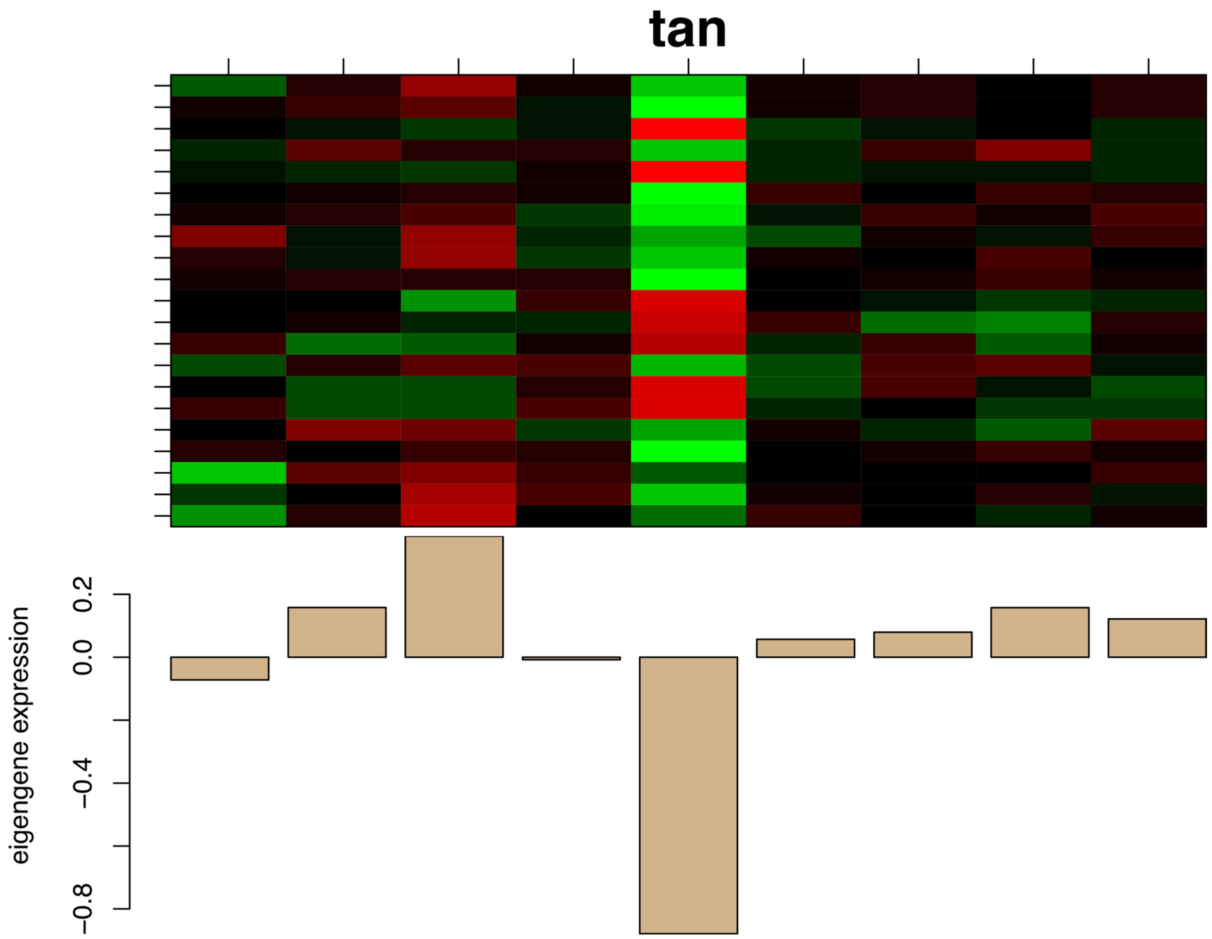

Supplementary Figure S2: The top row shows heatmap of the MEtan module genes (rows) across the sequencing samples (columns). The lower row shows the corresponding module eigengene expression values (y-axis) versus the same sequencing samples. Note that the module eigengene (ME) takes on low values in samples where a lot of module genes are under-expressed (green color in the heatmap). The ME takes on high values for samples where a lot of module genes are over-expressed (red in the heatmap). ME can be considered the most representative gene expression profile of the module.

Supplementary Table S1: Summary of lncRNA reads in the studied nine patients

Run	Left reads	Right reads	Overall Rate (%)
Liver cancer tissues			
BJ21	27,664,818	27,664,818	92.2
CQ105	28,107,814	28107814	93.4
CQ78<br>L6<br>L7<br>L8	 9,880,370<br>9,877,642<br>9,870,663	 9,880,370<br>9,877,642<br>9,870,663	 95.0<br>95.0<br>94.9
CQ83<br>L6<br>L7<br>L8	 10,051,023<br>10,049,393<br>10,040,724	 10,051,023<br>10,049,393<br>10,040,724	 94.3<br>94.3<br>94.2
CQ88<br>L6<br>L7<br>L8	 9,910,666<br>9,906,597<br>9,899,984	 9,910,666<br>9,906,597<br>9,899,984	 94.3<br>94.3<br>94.2
CQ94<br>L6<br>L7<br>L8	 9,880,727<br>9,877,760<br>9,873,873	 9,880,727<br>9,877,760<br>9,873,873	 94.1<br>94.1<br>94.0
CQ95	28,099,658	28,099,658	93.3
SN19	28,061,065	28,061,065	92.7
SN20	27,491,003	27,491,003	93.2
Liver cirrhosis tissues			
BJ21<br>L6<br>L7<br>L8	 9,734,425<br>9,690,494<br>9,684,916	 9,734,425<br>9,690,494<br>9,684,916	 93.1<br>93.9<br>93.8
CQ105	27,868,336	27,868,336	93.1
CQ78	27,968,582	27,968,582	94.0
CQ83	27,725,153	27,725,153	93.2
CQ88	28,131,234	28,131,234	94.0
CQ94<br>L6<br>L7<br>L8	 9,737,728<br>9,739,066<br>9,732,281	 9,737,728<br>9,739,066<br>9,732,281	 94.4<br>94.4<br>94.4
CQ95	27,372,445	27,372,445	92.4
SN19<br>L6<br>L7<br>L8	 9,604,207<br>9,601,696<br>9,598,458	 9,604,207<br>9,601,696<br>9,598,458	 93.8<br>93.8<br>93.6
SN20<br>L6<br>L7<br>L8	 9,723,916<br>9,720,567<br>9,711,098	 9,723,916<br>9,720,567<br>9,711,098	 94.0<br>94.0<br>93.9

Supplementary Table S2: Summary of mRNA reads in the studied nine patients

Run	Left reads	Right reads	Overall Rate (%)
Liver cancer tissues			
BJ21			
L6	9,483,516	9,482,518	93.3
L7	9,491,865	9,491,865	93.5
L8	9,512,124	9,512,124	93.5
CQ105			
L6	9,349,410	9,349,410	94.1
L7	9,358,641	9,358,641	94.2
L8	9,382,943	9,382,943	94.2
CQ78			
L6	9,444,486	9,444,486	93.6
L7	9,448,216	9,448,216	93.7
L8	9,467,979	9,467,979	93.7
CQ83			
L6	10,107,624	10,107,624	93.1
L7	10,106,349	10,106,349	93.1
L8	10,096,018	10,096,018	93.7
CQ88			
L6	9,521,369	9,521,369	95.5
L7	9,524,089	9,524,089	95.6
L8	9,546,112	9,546,112	95.6
CQ94			
L6	9,582,444	9,582,444	95.2
L7	9,593,706	9,593,706	95.2
L8	9,612,858	9,612,858	95.1
CQ95			
L6	9,559,175	9,559,175	96.0
L7	9,567,284	9,567,284	96.0
L8	9,590,578	9,590,578	96.0
SN19			
L6	9,436,377	9,436,377	94.6
L7	9,442,033	9,442,033	94.6
L8	9,469,773	9,469,773	94.6
SN20			
L6	9,646,014	9,646,014	96.0
L7	9,655,207	9,655,207	96.0
L8	9,679,183	9,679,183	96.0
Liver cirrhosis tissues			
BJ21			
L6	9,325,681	9,325,681	94.3
L7	10,486,601	10,486,601	94.3
L8	9,354,402	9,354,402	94.4
CQ105			
L6	9,534,194	9,534,194	95.3
L7	9,542,220	9,542,220	95.4
L8	9,564,240	9,564,240	95.4
CQ78			
L1	10,284,678	10,284,678	92.8
L2	10,262,860	10,262,860	92.8
L7	9,285,603	9,285,603	92.6
CQ83			
L6	10,081,302	10,081,302	91.6
L7	10,077,960	10,077,960	91.6
L8	10,106,576	10,106,576	91.6

CQ88			
L1	9,473,748	9,473,748	95.9
L2	9,462,705	9,462,705	95.9
L7	9,614,784	9,614,784	95.5
CQ94			
L6	10,039,383	10,039,383	92.3
L7	10,040,191	10,040,191	92.3
L8	10,024,769	10,024,769	92.3
CQ95			
L6	10,211,030	10,211,030	93.4
L7	10,210,318	10,210,318	93.3
L8	10,239,022	10,239,022	93.3
SN19			
L6	9,488,001	9,488,001	94.3
L7	9,494,712	9,494,712	94.3
L8	9,515,090	9,515,090	94.3
SN20			
L6	10,029,511	10,029,511	91.7
L7	10,028,781	10,028,781	91.6
L8	10,011,777	10,011,777	91.8

Supplementary Table S3: Top 10 GO terms for each cluster based on FDR

Cluster	Category	GO:ID	Terms	Count	%	*P* Value	FDR
B	CC	GO:0005576	extracellular region	66	27.73	1.81E–09	2.29E–06
	CC	GO:0044421	extracellular region part	41	17.23	7.21E–09	9.12E–06
	MF	GO:0005539	glycosaminoglycan binding	14	5.88	1.17E–07	1.68E–04
	MF	GO:0030247	polysaccharide binding	14	5.88	3.57E–07	5.12E–04
	MF	GO:0001871	pattern binding	14	5.88	3.57E–07	5.12E–04
	BP	GO:0007155	cell adhesion	29	12.18	5.72E–07	9.59E–04
	BP	GO:0022610	biological adhesion	29	12.18	5.89E–07	9.87E–04
	MF	GO:0030246	carbohydrate binding	20	8.40	9.12E–07	0.0013
	BP	GO:0002253	activation of immune response	10	4.20	6.99E–06	0.0117
	MF	GO:0048037	cofactor binding	15	6.30	1.57E–05	0.0225
C	CC	GO:0005576	extracellular region	12	5.56	3.13E–05	0.0316
E	CC	GO:0000786	nucleosome	11	16.18	6.79E–15	7.95E–12
	CC	GO:0032993	protein−DNA complex	11	16.18	1.78E–13	2.09E–10
	BP	GO:0006334	nucleosome assembly	11	16.18	2.41E–12	3.64E–09
	BP	GO:0031497	chromatin assembly	11	16.18	3.46E–12	5.23E–09
	BP	GO:0065004	protein−DNA complex assembly	11	16.18	5.48E–12	8.29E–09
	BP	GO:0034728	nucleosome organization	11	16.18	6.84E–12	1.03E–08
	BP	GO:0006323	DNA packaging	11	16.18	6.98E–11	1.05E–07
	BP	GO:0006333	chromatin assembly or disassembly	11	16.18	1.58E–10	2.39E–07
	CC	GO:0000785	chromatin	11	16.18	8.59E–10	1.01E–06
	CC	GO:0005694	chromosome	12	17.65	2.36E–07	2.77E–04

F	BP	GO:0007049	cell cycle	30	23.44	1.52E–13	2.39E–10
	BP	GO:0000279	M phase	20	15.63	2.85E–12	4.48E–09
	BP	GO:0022402	cell cycle process	23	17.97	1.05E–10	1.66E–07
	BP	GO:0022403	cell cycle phase	20	15.63	1.53E–10	2.40E–07
	BP	GO:0051301	cell division	17	13.28	4.51E–10	7.10E–07
	BP	GO:0007067	mitosis	15	11.72	7.69E–10	1.21E–06
	BP	GO:0000280	nuclear division	15	11.72	7.69E–10	1.21E–06
	BP	GO:0000087	M phase of mitotic cell cycle	15	11.72	9.74E–10	1.53E–06
	CC	GO:0044427	chromosomal part	18	14.06	1.27E–09	1.58E–06
	BP	GO:0048285	organelle fission	15	11.72	1.30E–09	2.04E–06

There was no significant GO term in the Cluster A and Cluster D.
Count: the number of genes belong to each term in a given gene list. %: the percentage of genes belong to each term in a given gene list. FDR: False discovery rate. BP: biological process, MF: molecular function, CC: cellular component.

Supplementary Table S4: The significant gene sets at $p < 0.05$ and FDR < 0.05

Gene signature	GSSize	ES	ES.pos	*P*	FDR	FWER
BCAT_GDS748_DN	40	0.569	4332	0.005	0	0.246
BCAT.100_UP.V1_DN	29	0.572	2973	0.041	0	0.233
RPS14_DN.V1_DN	151	0.539	3819	0	0	0.462
CORDENONSI_YAP_CONSERVED_SIGNATURE	52	0.569	3090	0.006	0	0.248
KRAS.50_UP.V1_DN	20	0.571	1118	0.004	0	0.235

GSSize: gene set size; ES: enrichment score; ES.pos: ES. Position; FDR: false discover rate; FWER: Family−wise error rate.

Supplementary Table S5: The clinical features of 78 HCC patients for validation

ID	Sex	Age (y)	HBV DNA	HBsAg	HBeAg	AFP1(ng/ml)
CQ100	Male	61	3.51E + 05	+	+	86638
CQ102	Male	79	< 1.0E + 03	−	−	764.8
CQ125	Female	56	3.03E + 05	+	−	3.2
CQ126	Male	44	1.73E + 03	+	+	1141
CQ128	Male	49	1.51E + 04	+	−	2.15
CQ130	Male	59	4.36E + 03	+	−	8.38
CQ131	Male	42	3.06E + 04	+	+	363.3
CQ132	Male	58	3.24E + 07	+	−	198.39
CQ133	Male	30	1.48E + 05	+	+	> 484000
CQ134	Male	41	NA	+	+	467.88
CQ136	Male	64	3.06E + 04	+	−	6.42
CQ137	Male	32	< 1.0E + 03	+	−	1.04
CQ138	Male	40	3.42E + 04	+	−	277.2
CQ141	Male	55	< 1.0E + 03	+	−	200
CQ143	Female	52	5.51E + 05	+	+	75620
CQ145	Male	37	< 1.0E + 03	+	−	797.5
CQ146	Male	55	6.85E + 03	+	−	373.8
CQ148	Male	49	NA	+	−	6837
CQ149	Male	48	< 1.0E + 03	+	−	1.37
CQ153	Male	35	NA	+	−	1.93
CQ154	Male	50	4.89E + 03	+	−	1.45
CQ155	Male	55	6.85E + 03	+	−	32.46

CQ157	Male	35	< 1.0E + 03	+	+	1144
CQ158	Male	36	< 1.0E + 03	+	−	45.68
CQ160	Male	71	< 1.0E + 03	+	−	1.08
CQ161	Male	57	1.61E + 05	+	+	3421
CQ163	Male	52	2.91E + 05	+	−	97.65
CQ175	Male	27	< 1.0E + 03	+	−	232.51
CQ176	Female	38	< 1.0E + 03	+	−	179.44
CQ177	Male	56	7.82E + 03	+	−	359.6
CQ178	Male	68	1.25E + 03	+	−	5.86
CQ179	Male	44	2.98E + 07	+	+	NA
CQ181	Female	50	1.88E + 05	+	+	18.72
CQ182	Male	43	7.43E + 04	+	−	3588
CQ183	Male	56	3.98E + 05	+	−	7.15
CQ184	Male	19	< 1.0E + 03	+	+	194.7
CQ185	Male	43	1.40E + 04	+	−	3.74
CQ186	Male	49	2.36E + 04	+	−	1210
CQ187	Male	58	NA	+	−	15.81
CQ190	Male	50	< 1.0E + 03	+	−	25.45
CQ191	Male	50	NA	+	−	2.26
CQ192	Male	64	< 1.0E + 03			30.65
CQ193	Male	77	< 1.0E + 03	−	−	3.31
CQ194	Male	57	< 1.0E + 03	+	−	866.1
CQ195	Male	66	< 1.0E + 03	−	−	245.8
CQ76	Male	58	< 1.0E + 03	+	−	8.19
CQ80	Male	31	NA	−	−	1.85
CQ81	Male	62	< 1.0E + 03	+	+	24.26
CQ89	Male	62	< 1.0E + 03	−	−	5.32
CQ91	Male	38	2.08E + 03	+	+	848.6
CQ93	Female	40	< 1.0E + 03	+	+	143.4
CQ96	Male	49	4.40E + 04	+	−	2.1
CQ99	Male	58	4.60E + 06	+	+	2.49
HN07	Male	66	2.92E + 04	+	+	> 1210
HN10	Female	37	8.77E + 03	+	+	172.4
HN12	Male	59	2.00E + 06	+	+	432.7
HN13	Female	56	3.23E + 05	+	+	> 1210
HN15	Male	66	1.14E + 05	+	+	> 1210
HN18	Male	59	< 5.00E + 02	+	+	6.53
HN20	Male	31	NA	+	+	> 1210
HN22	Male	72	NA	+	+	29.66
HN25	Male	51	< 5.00E + 02	+	+	7.07
HN30	Male	51	< 5.00E + 02	+	+	2.46
SN12	Male	56	NA	+	−	1210
SN13	Male	36	1.06E + 03	+	−	1210
SN15	Male	62	1.15E + 05	+	−	1210

SN16	Male	43	< 5.00E + 02	+	−	5.29
SN17	Male	47	< 5.00E + 02	+	−	1210
SN19	Male	50	< 5.00E + 02	NA	NA	186.1
SN20	Male	55	1.49E + 05	+	−	4.35
SN23	Male	49	NA	+	−	8.22
SN25	Male	61	2.32E + 04	+	−	9.63
SN26	Male	49	NA	+	−	1210
SN27	Female	41	1.50E + 03	+	−	868.4
SN28	Male	61	9.24E + 05	+	−	484.8
SN29	Male	48	< 5.00E + 02	+	−	5.09
SN30	Male	69	< 5.00E + 02	+	−	89.98
SN31	Female	50	< 5.00E + 02	+	−	1210

[1]AFP = Alpha fetoprotein.
